# Self-assembly growth of electrolytic silver dendrites

**DOI:** 10.1038/s41598-022-08586-2

**Published:** 2022-03-16

**Authors:** Wen-Chieh Tsai, Kwang-Lung Lin

**Affiliations:** Department of Materials Science and Engineering, National Cheng Kung University, No. 1, University Road, Tainan, 70101 Taiwan, ROC

**Keywords:** Molecular self-assembly, Molecular self-assembly

## Abstract

The atomic level assembly of silver dendrite has never been disclosed despite the numerous studies published on fractal dendrite structures. We report for the first time an HRTEM investigation of the formation of atomic embryos (< 5 nm) and the self-assembly of atoms on an atomic plane of the tip of a dendrite arm. The mechanism of dendrite formation proceeds via the sequence of amorphous embryos aggregates (5–10 nm), nuclei, crystallites (10–20 nm), dendritelets (50–100 nm) and submicron dendrite protypes. The atomic plane is an entirely atomic-level zig-zag structure with d-spacing kink steps. The zig-zag structure triggers the self-assembly of atoms and thus directional growth to produce a dendrite arm with a high aspect ratio.

## Introduction

Silver dendrite has been found in various applications in sensors^1^, catalysis^2^, surface enhanced Raman scattering^3^, and electronics^4,5^, among others. Silver dendrite also exhibits better sintering and percolation performance in electric conductive composites than silver flakes^4^. The methods available for producing silver dendrite include replacement reactions^6^, electrochemical^7–9^, solvothermal^10^, oxidation reduction^4^, wet chemical^11^, and template reduction^12^ methods, etc.

Dendrites generally grow from a primary dendrite stem to secondary dendrite and tertiary dendrite arms. The fractal dendrite growth induced by electrochemical migration has been simulated and analyzed^13^. Each portion of the dendrite structure is single crystal in nature^14^, as also revealed in the TEM image (Fig. S1a) and the electron diffraction (Fig. S1b). The secondary dendrite arm can grow vertically or slanted at around a 60° angle relative to the primary dendrite stem (Fig. S1a)^15^. The dendrite shape of electrodeposited silver structures has been determined based on deviations from equilibrium^16^. The morphology of silver dendrite is governed by the competition between thermodynamic and kinetic factors^17^. At a short reaction time, the concentration gradient dominates the growth and thus controls kinetic factors. At a longer reaction time, the relaxation of small grains occurs, which gives rise to a hexagonal plate head. The growth mechanism of silver dendrite, with a final hexagonal dendrite head^7,18^, has been attributed to oriented attachment^6,7^. The aggregation and coalescence of nano particles may occur via oriented attachment^19^ and may lead to attachment to the dendrite tip, which accelerates dendrite growth^20–22^.

As mentioned above, numerous studies have discussed the growth behavior of fractal dendrite. These previous studies mostly focused on discussing the morphology of dendrite, including the hexagonal dendrite head and the slanted structure. However, there has been little discussion of the origin of dendrite formation and the directional growth behavior of a dendrite arm with a high aspect ratio. High aspect ratio morphology is indicative that the growth rate of dendrite is much faster in the longitudinal direction than in the circular radial direction of the dendrite arm. We report here for the first time the aggregation of atoms to form nano embryos and thereafter the sequential formation up to dendrite using HRTEM (High Resolution Transmission Electron Microscopy). The findings of this study also reveal the existence of an atomic plane with a level of d-spacing zig-zag kink step on the dendrite tip. The self-assembly of atoms on a high surface energy zig-zag atom plane contributes to the formation of a dendrite arm with a high aspect ratio.

The selection of silver dendrite for investigation provides not only an example but also a practical need. The growth of particles via self-assembly process may give rise to various morphologies. The spectacular morphology of silver dendrite implies a complicate growth mechanism. A detail investigation of the growth mechanism will provide better understanding to most of the self-assembled particle growth behavior. The growth of dendrite tip reveals the self-assembly of atoms and provides the advantage over other types of particles for easy targeting the location for HRTEM investigation. The repeating formation of dendrite tip provides an excellent location for investigating the growth behavior of self-assembled particles.

## Methods

**Figure 1:**
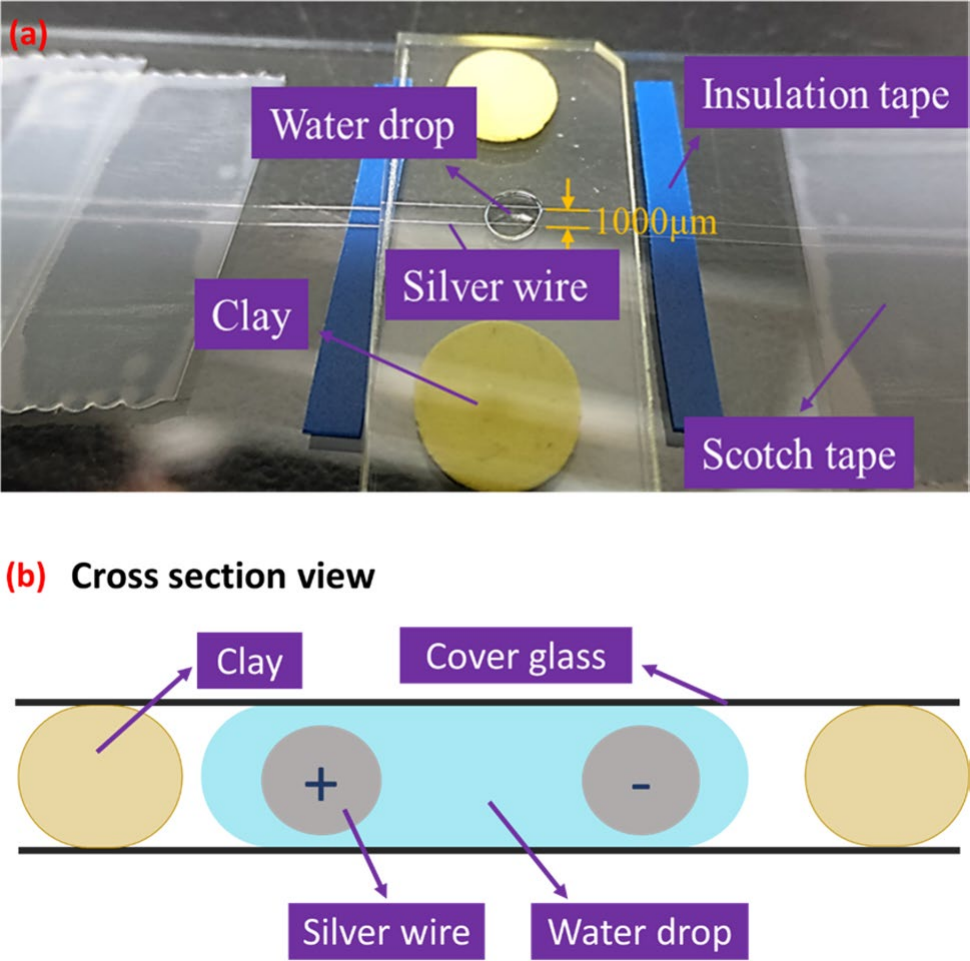
(**a**) The setup of the electrolytic process. (**b**) The silver wires were arranged at a 1000 µm distance from each other and fixed with Scotch tape. The cover glass plate on top of the silver wires was fixed on the sample holder with clay to maintain the stand-off. A drop of deionized water underneath the glass plates surrounded the two silver wires. The two silver wires served as the cathode and anode, respectively. The waster drop region between the two silver wires was observed under an optical microscope during electrical current stressing. A CCD camera was equipped to take a video of the experiment.

In the present study, an electrolytic method (S1 and Fig. 1) was applied for the purpose of preparing nano silver dendrites in pure water media using commercial silver alloy wires as the cathode and anode. The experiment presents a novel method for manufacturing silver nano particles and dendrites without chemical contaminants. The anodic wire became dull soon after the electric current was applied, indicating the occurrence of oxidation. The silver ions released from the anodic silver wire under electrical potential migrated towards the cathodic wire and were reduced thereon. The electroconvection induced fluid circulation^23^ was reflected by the flowing particles stream (Fig. S2). The silver particle stream became visible in the media within 5 s of current stressing. The particle stream moved clockwise or counterclockwise, indicating the existence of various types of fluid circulation in the water media. The video frames shown in Fig. S2 indicate that the silver dendrites growing from the cathodic wire swung and reached out to attract the suspended particulates (to be discussed later) in circulation which assisted the dendrite growth, possibly due to an oriented attachment mechanism^6^. The attraction of the suspended particulates is a relatively macroscopic contact visible only under an optical microscope in this study, as compared with the microscopic atomic assembly described later. However, the phenomena of self-assembly of atoms, the formation and existence of embryos, embryo aggregates, crystallite nuclei, crystallites, and dendritelets (these terms are discussed in the following paragraph) in the water media have never been disclosed in detail. We report and discuss for the first time the formation and progress of the various levels of nano products during the growth of electrolytic silver dendrites.

The electrolytic products were washed off the cathodic wire with ethanol and placed on a petri dish for sampling. The ethanol suspension was treated with an ultrasonic vibration for 20 s and then transferred onto a copper grid with a pipet for the HRTEM investigation after drying. The formation of electrolytic products and the dendrite growth took place continuously during the electrolytic process. Thus, the electrolytic products collected and observed under TEM contained all levels of the products produced from the very beginning of atomic aggregation (the embryo) and self-assembly of atoms to the fractal dendrites. A detailed inspection and analysis of the products will hopefully disclose the progress of the dendrite formation.

## Results and discussions

**Figure 2:**
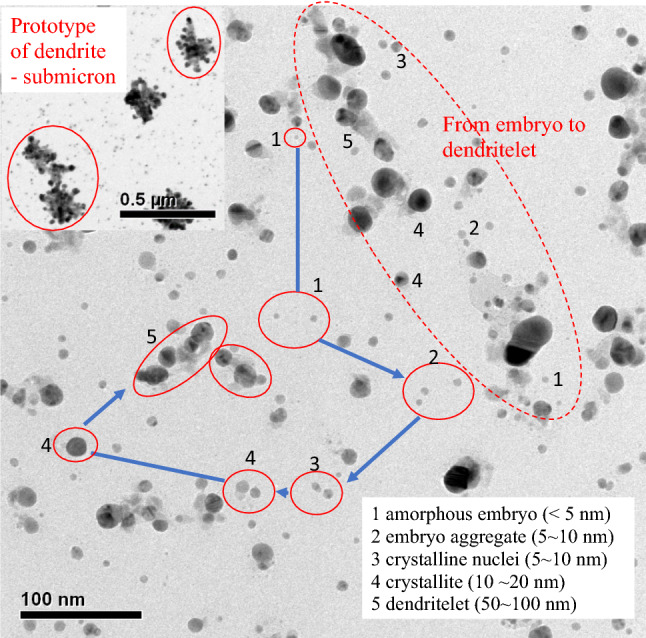
The sampled electrolytic products reveal the history or mechanism of the dendrite formation. The silver ions produced from the anode wire due to the electrolytic potential were reduced to silver atoms at the cathodic wire. The silver atoms aggregated to form amorphous embryos, where 1 had a diameter of less than 5 nm. The nano embryos approached each other to form the embryo aggregates, where 2 had diameters of 5–10 nm. The embryos and the embryo aggregate further coalesced and transformed into crystalline nuclei, where 3 had a diameter ranging from 5 to 10 nm. The crystalline nuclei when nourished by embryos, embryo aggregates, and nuclei grew into crystallites, 4 with a diameter of 10–20 nm. The crystallites exhibited a lattice orientation and thus became fully dark upon growth. The agglomeration of crystallites tended to reorient them to shape the dendritelets, where 5 had a diameter of 50–100 nm. The broken helical circle is a summary of the collection, which further demonstrates the stepwise progress from amorphous embryos (1) to the ultimate dendritelets (5). The aggregation of the dendritelets gave rise to the formation of the dendrite prototype, as shown in the inset.

The silver ions released from the anodic wire were soon reduced to silver atoms at the cathodic wire. Most of the silver atoms produced were carried away from the cathodic wire by the circulation flow. It is thus inferred that the water media was enriched with silver atoms within a few seconds after the electric potential was applied. This fluid circulation assisted in the aggregation of silver atoms to form the amorphous embryos, shown as 1 in Fig. 2. The spherical embryos were less than 5 nm in diameter. The nano embryos approach each other as a result of circulation and the van der Waals force to form the 5–10 nm embryo aggregates, shown as 2 in Fig. 2. Subsequently, the embryos and the embryo aggregated further, coalesced, and converted to 5–10 nm crystalline nuclei, shown as 3 in Fig. 2, as evidenced by the darker dotted appearance of the particulate. The system energy state was lowered from the high energy status of amorphous embryos and embryo aggregates to the more stable crystalline nuclei. The nuclei began to exhibit a somewhat ordered structure. The next step was the growth of the crystalline nuclei to the crystallite, shown as 4 in Fig. 2, when nourished by the silver atoms, embryos, and embryo aggregates. The crystallites were 10–20 nm in diameter. This is visible from the crystallite 4 left to the crystalline nuclei 3 in Fig. 2 where a pale nano embryo approaches a dark crystallite particle. The crystallites exhibit lattice orientation and thus become fully dark upon growth, as shown by the 4 in the lower left of Fig. 2. The agglomeration of crystallites tended to lead to reorientation and to shape the dendritelets, see 5 in Fig. 2, which ranged in size from 50 to 100 nm. The broken helical circle in the upper right of Fig. 2 is a summary, which further demonstrates the progress from amorphous embryo 1 to embryo aggregate 2, crystalline nuclei 3, crystallite 4 and ultimately to dendritelets 5. The aggregation of the dendritelets along with the nourishing from 1 to 4 produced the dendrite prototype, as presented in the inset of Fig. 2. A dendritelet is regarded as the basic unit for the dendrite prototype. The submicron dendrite prototype distributed throughout the water media. The dendrite prototype is believed to be in the prio-state when forming the fractal dendrite. The primary dendrite stem reaches out to rapidly grab the dendritelets as well as the dendrite prototype. The attraction of the dendritelet and the dendrite prototype from the water media expedites the growth of the fractal dendrite from the cathodic silver wire. It is believed that the electrical potential on the tip of the dendrite in the primary dendrite stem facilitates the attraction of the suspended dendritelets and prototypes.

The formation of an amorphous embryo is regarded as the origin of the self-assembly scheme producing the fractal dendrite. The self-assembly scheme illustrated in Fig. 2 is intended to produce suspended products. The other self-assembly scheme of the silver atoms occurs on the dendrite arms, which also expedites growth and governs the morphology of the dendrite arm.

**Figure 3:**
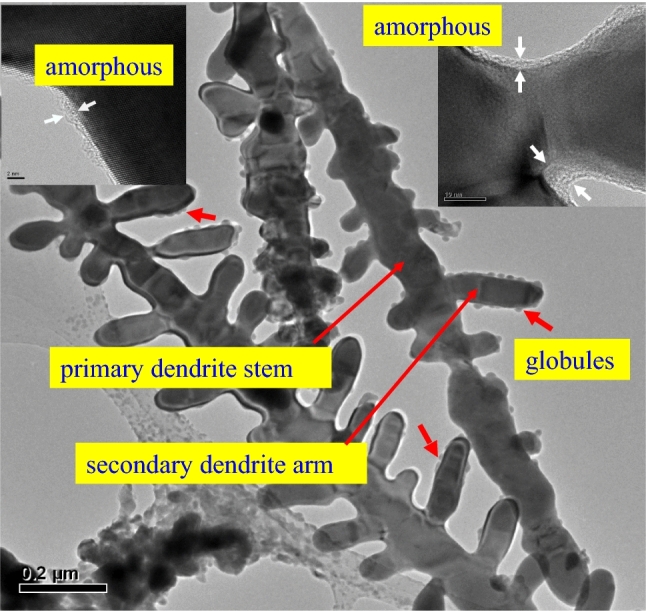
The dendrites were covered by a thin nano amorphous covering, with a thickness of less than 10 nm, as indicated by the arrows shown in the insets. Many of the dendrites had particulate products with dimeter of less than 50 nm adhered to them. The nano particulate attachments existed in the surrounding dendrite arm surface as well as on the dendrite tip. The high aspect ratio structure delineates the preferential growth of the dendrite arm in the longitudinal direction rather than in the radial direction. This indicated that the nano globules adhered to the surface of the dendrite arm contributed little to the growth of the dendrite.

It has been reported that the dendrite front and even the dendrite skin exhibit a thin amorphous layer at the nanometer scale^15,18,24^, which indicates the accumulation of reduced atoms. The insets in Fig. 3 reveal that there is a thin nano amorphous covering as shown by the arrow along the dendrite arm down to the concave connection between the primary dendrite stem and the secondary dendrite arm. The thickness of the amorphous covering here is smaller than 10 nm. Figure 3 further evidenced the adherence of globules smaller than 50 nm to the dendrite, as pointed out with short red arrows. The nano globules were attracted from the water media by either the electrical potential or the van der Waals force. The nano attachments were adsorbed on the dendrite arm skin surface as well as on the dendrite tip. Nevertheless, the directional growth of the dendrite in the longitudinal direction of the dendrite arm indicates that the nano globules adhered on the tip of the dendrite arm were the main contributors to the growth of the dendrite arm.

**Figure 4:**
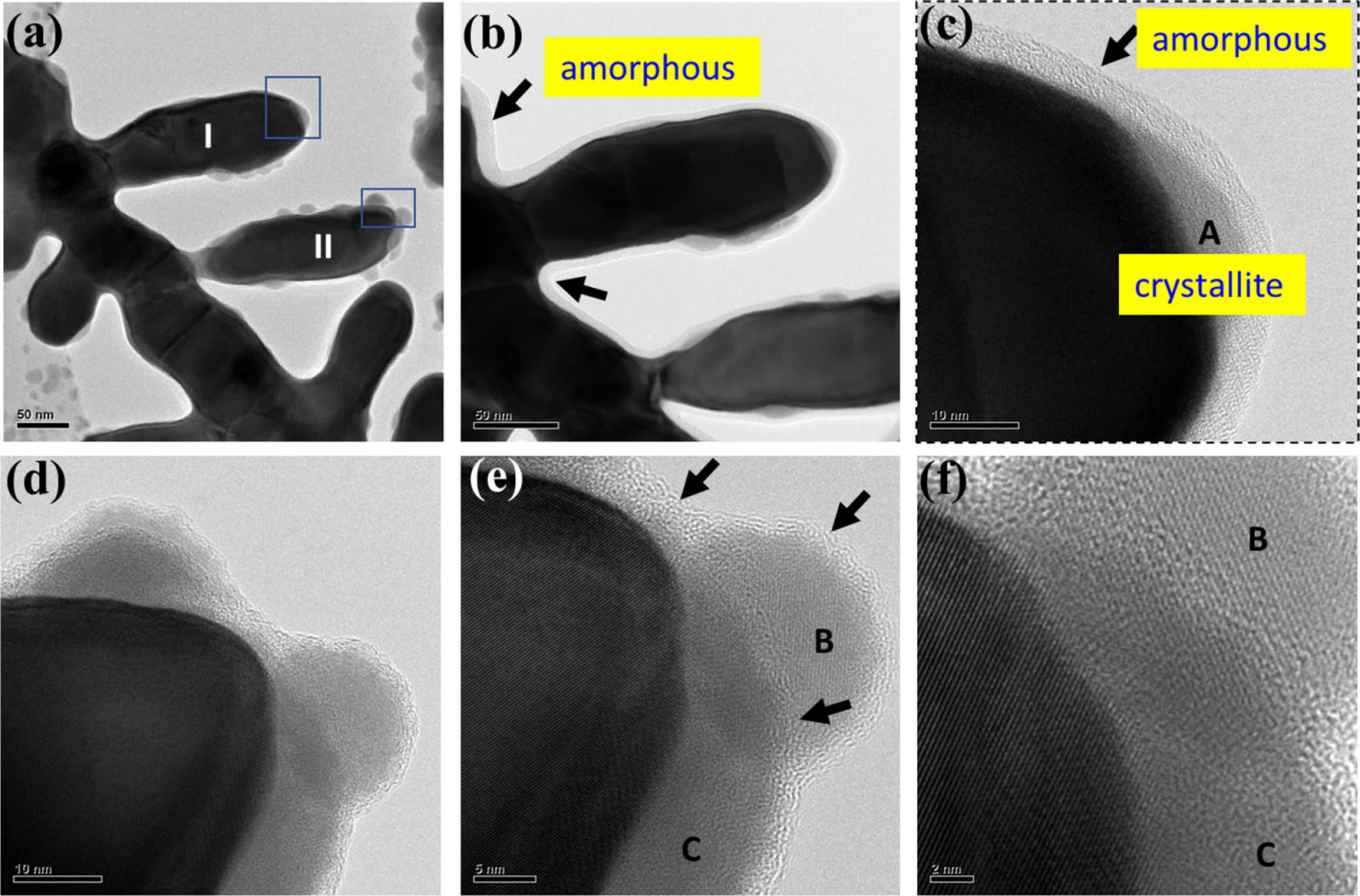
(**a**) The globules with a diameter same as crystallite (10–50 nm) attached to the arm surface of dendrite arm I and arm tip of dendrite arm II. The dendrite was entirely covered by an amorphous nano covering, as indicated by the arrows in (**b**) and (**c**). (**c**) A nano crystallite A lay on the tip front of dendrite arm I and embedded in the rim of the amorphous nano covering. Crystallite A exhibited small contact angle of less than 45°, with the underneath tip front exhibiting a longer base line than its height. (**d**) Two nano globules adhered on the tip of dendrite arm II. Globules B and C were covered by an amorphous nano covering less than 5 nm thick, as shown in (**e**). The amorphous nano covering between B and C distinguishes the two separate crystallites. The lattice front of the lower left image in (**f**) shows the lattice front received reoriented crystallites from crystallite C. The adsorption of 10–20 nm dimeter crystallites on the dendrite tip facilitated and accelerated the dendrite arm growth via an oriented attachment.

The dendrite arms labeled as I and II shown in Fig. 4a have nano-globules attached. The globules have the same size as that of crystallite (10–20 nm). The magnified image Fig. 4b clearly shows the existence of an amorphous nano covering, as indicated by the arrows, surrounding the entire dendrite structure. The tip front of dendrite arm I, Fig. 4c, further evidences a nano crystallite, A, lying on the tip front while also embedded in the rim of the nano amorphous covering. The crystallite A appears to have a small contact angle of less than 45^o^ from the underneath side of the tip front. The contact interface has large base as compared with the height of A. Figure 4d–f present various magnified tip images of dendrite arm II shown in Fig. 4a, where two globules can be seen on the dendrite tip. Similarly, the nano amorphous covering, as shown with an arrow in Fig. 4e, with a thickness of less than 5 nm is observable surrounding the curvature of the dendrite tip. The two crystallites (B and C) have a lattice orientation, as shown in Fig. 4e and f. The presence of an amorphous nano covering (Fig. 4e) between B and C distinguishes the two separate crystallites. The darker images in the lower portion of B and upper portion of C signify overlapping images of these two crystallites. It is believed that the nano crystallites B and C serves as the prior state that assisted in the growth of the dendrite arm outwards from the dendrite tip. Crystallite C was shaped like A in Fig. 4c, lying on the tip front with a small contact angle. The nano crystallite reorients itself and aligns with the lattice orientation of the dendrite, which is known as oriented attachment^6,7^, to lower the interfacial energy. The prior formation and attachment of the nano crystallites within the amorphous covering is one of the intermediate states for the dendrite arm growth resulting from the assembly of atoms. The lattice front of the lower left image shown in Fig. 4f is an extension of the lattice structure towards the crystallite C. The lattice front receives the reoriented crystallites embedded in the amorphous covering. Basing on the observations shown in Figs. 2 and 4, the crystallites (4 in Fig. 2) with thicknesses ranging from 10 to 20 nm serve as an important pre-state for dendrite growth in addition to the seed for the formation of dendritelets (see 5 in Fig. 2). The adsorption of crystallites on the tip facilitates and expedites the growth of the dendrite arm via an oriented attachment process. The atom plane structure discussed here is believed to also benefit the adsorption of crystallites on the tip of the dendrite arm and thus favors the directional growth of the dendrite arm.

**Figure 5:**
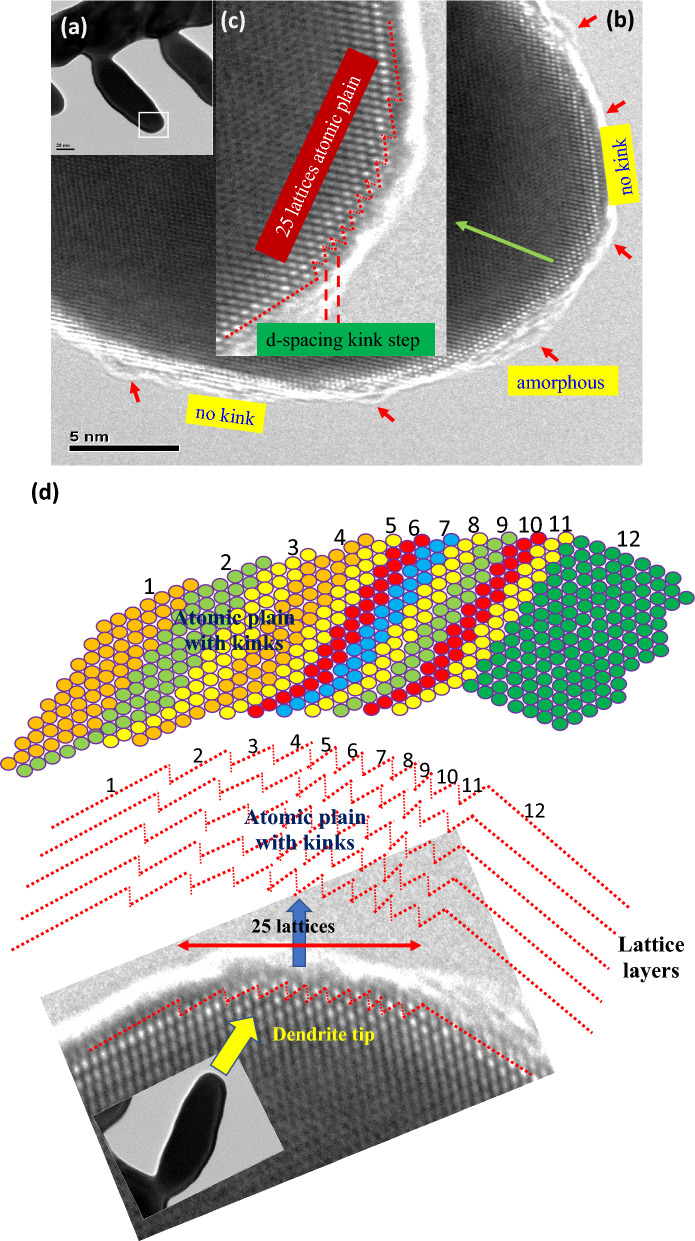
(**a**) The dendrite arm without globule attachment still exhibited growth behavior indicative of a high aspect ratio. The growth behavior was closely related to the high surface energy of the tip area, which enabled rapid adsorption of atoms. (**b**) The high-resolution TEM image reveals the rim of the amorphous nano covering surrounding the curved region of the arm tip, as indicated by red arrows. The upper right top and lower left bottom show straight linear lattice structure with no kinks. (**c**) The tip end shows zig-zag kink steps for which the kink step has a one d-spacing. The zig-zag structure area is comprised of 25 lattices forming an atomic plane when extended two dimensionally. (**d**) Top view simulation sketch presents the 25 lattices atomic plane on the dendrite tip with an average roughness (Ra) of one d-spacing and the fronts of the lattice segment from the atomic plane. The kinks provided the high surface energy for atom adsorption that favored the growth of the secondary dendrite arm in the longitudinal direction. The numbered planes show extending segment of the underneath lattice plane that form the zig-zag structure. The round curvature of the dendrite tip is formed with short segments, planes 3–11.

The growth of the dendrite arm also takes place directly from self-assembly of atoms on the dendrite tip without contributions from globule attachment. The upper left inset Fig. 5a presents a secondary dendrite arm without globule attachment. The high aspect ratio of the secondary dendrite arm implies favorable growth of the dendrite arm from the tip rather than from the round-arm surface. The diameter of the dendrite arm remains somewhat the same. Figure 5b presents an HRTEM image of the confined area of the tip of the dendrite arm shown in Fig. 5a. The red arrows point out the rim of the amorphous nano covering. The high-resolution HRTEM image reveals the lattice structure on the tip. The inset Fig. 5c presents a further enlarged image of the very front of the tip. The dash red line illustrates the profile of the very front lattice rows. The profile shows a gathering of dense stair kinks at the tip front. It is apparent that the kinks are formed as a result of the stacking of lattice planes with various dimensions. The upper right and the lower left portions of the link exhibit relatively long straight lattice structures without stair kinks. The bottom edge of Fig. 5b also shows a relatively long straight lattice front without a kink structure. It is evident from the cross-sectional curvature that the tip of the dendrite arm has a high surface energy zig-zag structure. The kink is located at the end of the lattice plane. The step size of the zig-zag structure has the same dimension as that of the d-spacing of the lattice structure, as visualized in Fig. 5c. If one visualized a stereo top view, the dendrite tip can be regarded as comprising at least one atomic plane enriched with kinks, as illustrated by the sketch in Fig. 5d. The numbered planes show the stacking sequence. It shows twelve planes with ten steps (number 2 to 11). Each step was formed as resulted from the extending segment of the underneath plane. The steps jointly form the zing-zag structure. The combination of short segments, planes 3–11, gives rise to the round curvature dendrite tip. The kinks reflect a surface roughness of only one d-spacing dimension. The diameter of the examined circular atomic plain is approximately 25 d-spacing as shown in Fig. 5c. Accordingly, the area of the atomic plane was estimated to be 160 π × (d-spacing)^2^. The atomic plane was covered by the amorphous rim, which is the preliminary gathering of atomic resources. The atoms within the meta-stable amorphous rim can self-assemble easily on the kinks of the atomic plane, as pointed out with the blue arrows. The unclear dots in the figure reflect the self-assembly of atoms. It is believed that the high surface energy kink structure benefits the adsorption of atoms from the amorphous nano covering. A combination of an abundant supply of atoms from the amorphous nano covering with the large surface area comprising high energy zig-zag stair kinks will result in fast self-assembly of atoms. Consequently, the fast self-assembly of atoms on the atomic plane gives rise to the directional growth of the high aspect ratio secondary dendrite arm.

Looking back at the images shown in Fig. 4c and f, it is likely that there also were some atomic planes on the dendrite tip. The plane diameter of the underneath crystallite A on Fig. 4c was around 20 nm. The atomic plane herein definitely enhances the attachment of crystallite A. The adhering of A brought to the tip a relatively large number of atoms required for dendrite growth. It is thus inferred that the high surface energy atomic plane greatly facilitated the adherence of the crystallite to the dendrite tip and thus expedited the directional growth of the dendrite arm. In view of the content shown in Figs. 4 and 5d, it is emphasized herein that the formation of crystallites, 4 in Fig. 2, and the atomic plane, as shown in Fig. 5c plays an important role in expediting the directional growth of the dendrite arm in the longitudinal direction and thus the formation of a dendrite arm with a high aspect ratio.

The mechanism of the dendrite growth indicates that the dendrite morphology appears after the stage of the “crystallite” formed. The “dendritelet” starts to form as resulted from the aggregation of the 10–20 nm “crystallite”. There are many other dendrite metal powders mentioned in the literature. For instances, electrolytic dendritic copper powder is available commercially, while the lithium dendritic powder was produced at the Li powder anode^25,26^. It is believed that the mechanism revealed for the silver dendrite is common and a breakthrough understanding of all dendritic powder formation and growth. The mechanism implies that it is possible to produce non-dendritic particle by cutting off the formation of dendritelet formation. One of the possible ways is to hinder the aggregation of the “crystallite” to avoid the formation of the “dendritelet”. By doing so, a very fine nano dimension of non-dendritic particle could be produced. The reveal of the mechanism is believed to inspire further technology development of particle morphology control.

## Supplementary Information

Supplementary Information.
