# Publisher Correction: Self-assembly growth of electrolytic silver dendrites

**DOI:** 10.1038/s41598-022-12065-z

**Published:** 2022-05-17

**Authors:** Wen‑Chieh Tsai, Kwang‑Lung Lin

**Affiliations:** Department of Materials Science and Engineering, National Cheng Kung University, No. 1, University Road, Tainan, 70101 Taiwan, ROC

Correction to: *Scientific Reports* 10.1038/s41598-022-08586-2, published online 16 March 2022

The original version of this Article contained an error in Figure 5 where Figure 5 (d) was omitted. The original Figure 5 and accompanying legend appear below.

**Figure 5:**
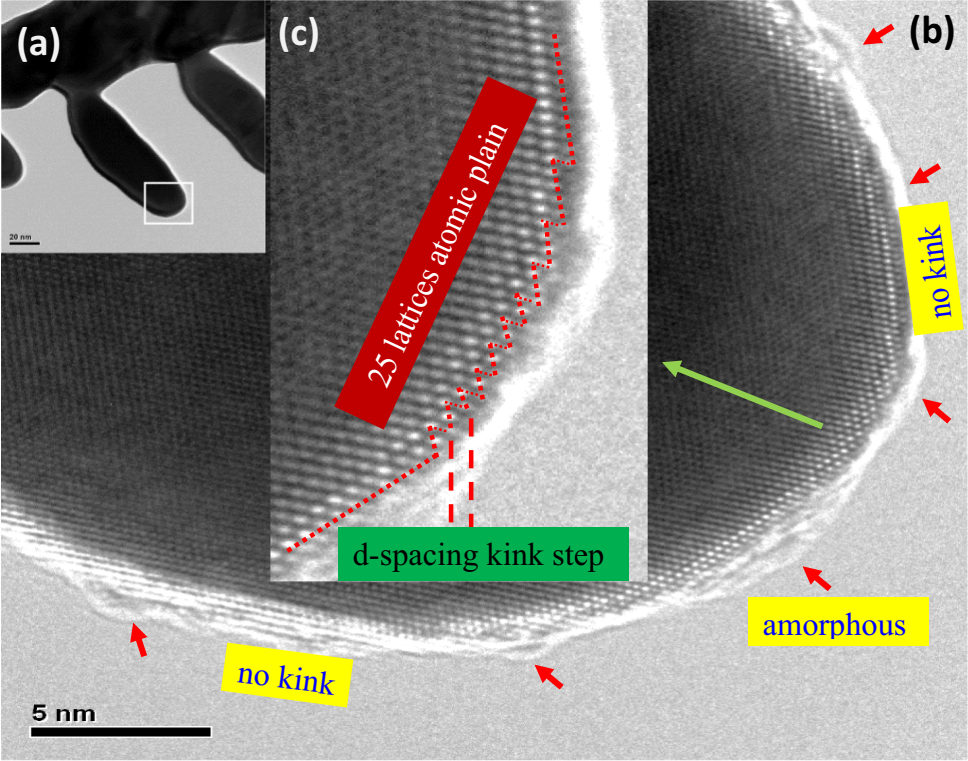
The caption to be typeset alongside it: (**a**) The dendrite arm without globule attachment still exhibited growth behavior indicative of a high aspect ratio. The growth behavior was closely related to the high surface energy of the tip area, which enabled rapid adsorption of atoms. (**b**) The high-resolution TEM image reveals the rim of the amorphous nano covering surrounding the curved region of the arm tip, as indicated by red arrows. The upper right top and lower left bottom show straight linear lattice structure with no kinks. (**c**) The tip end shows zig-zag kink steps for which the kink step has a one d-spacing. The zig-zag structure area is comprised of 25 lattices forming an atomic plane when extended two dimensionally. (**d**) Top view simulation sketch presents the 25 lattices atomic plane on the dendrite tip with an average roughness (Ra) of one d-spacing and the fronts of the lattice segment from the atomic plane. The kinks provided the high surface energy for atom adsorption that favored the growth of the secondary dendrite arm in the longitudinal direction. The numbered planes show extending segment of the underneath lattice plane that form the zig-zag structure. The round curvature of the dendrite tip is formed with short segments, planes 3–11.

The original Article has been corrected.

