# Supplementary material for: Self-assembly growth of electrolytic silver dendrites
Source: Sci Rep. 2022 Mar 16;12:4479. doi: 10.1038/s41598-022-08586-2 (PMC8927591; doi:10.1038/s41598-022-08586-2)
Supplement: Supplementary file 1 — Supplementary Information. [file 41598_2022_8586_MOESM1_ESM.docx]

**Supplementary Materials**

**S1. Materials and Methods**

Silver dendrites were produced with electrolytic method in deionized water. The supplied commercial silver wire contained fractional alloying elements Pd and In. The detailed composition of the silver wire was not released for commercial reasons. The inclusion alloy did not lead to an observable effect on the dendrite growth in the present study. Two silver wires were arranged to maintain a 1 mm distance on a microscope glass plate. The silver wires were attached with double-sided Scotch tape to maintain the distance and further secured on the sample holder (Figure 1). The glass plate was fixed on the sample holder with clay to maintain the stand-off. A drop of deionized water was applied as the electrolyte between the two silver wires. The region of the water drop between the two silver wires was observed under an optical microscope during the electrical current stressing. Deionized water was used to avoid any contamination or ion source other than that from the silver wire. The two silver wires served as the cathode and anode, respectively, during the electrolysis process. The electrolysis was conducted with an electrical current of 5 volts and 0.5 mA. The anodic silver wire gradually dissolved, yielding silver ions migrating toward and being reduced at the cathodic wire. The ion migration in the electrical field induced vigorous electrolyte circulation revealed by the flowing of the stream of particles observed in the microscopic video (available upon request) taken above the water drop. The electrolytic products were washed off the cathodic wire with ethanol and placed on a petri dish for sampling. The ethanol suspension was treated with ultrasonic vibration for 20 sec and then transferred onto a copper mesh with a pipet for high resolution TEM (HRTEM) investigation after drying.


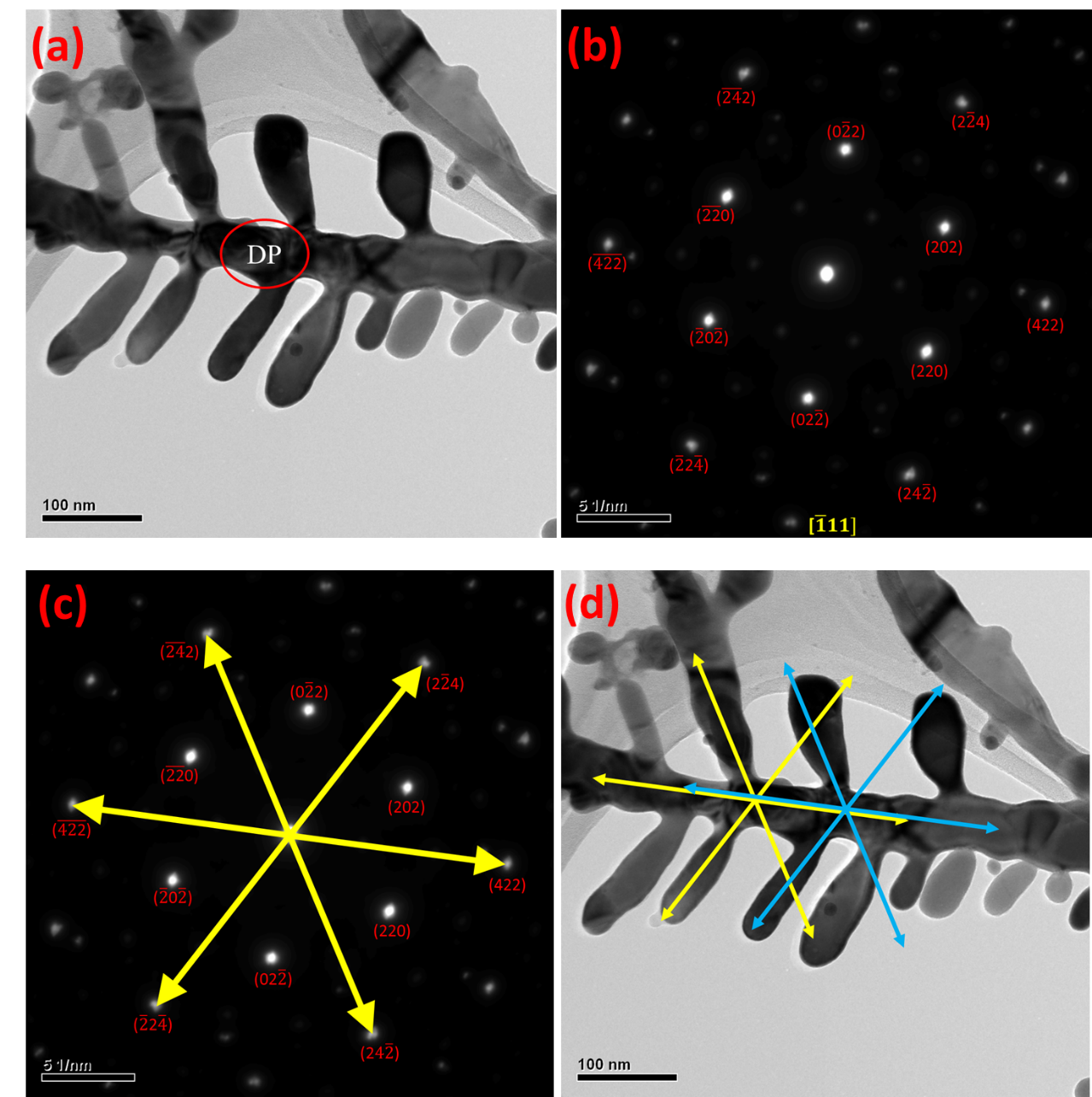


Figure S1 (a) The primary dendrite stem and secondary dendrite arm structures with a slanted angle arrangement of approximately 60^o^. Both perfect and imperfect secondary dendrites were observed on the same primary dendrite stem. (b) The electron diffraction of the primary dendrite stem (position DP) showing the crystalline structure of the silver dendrites.


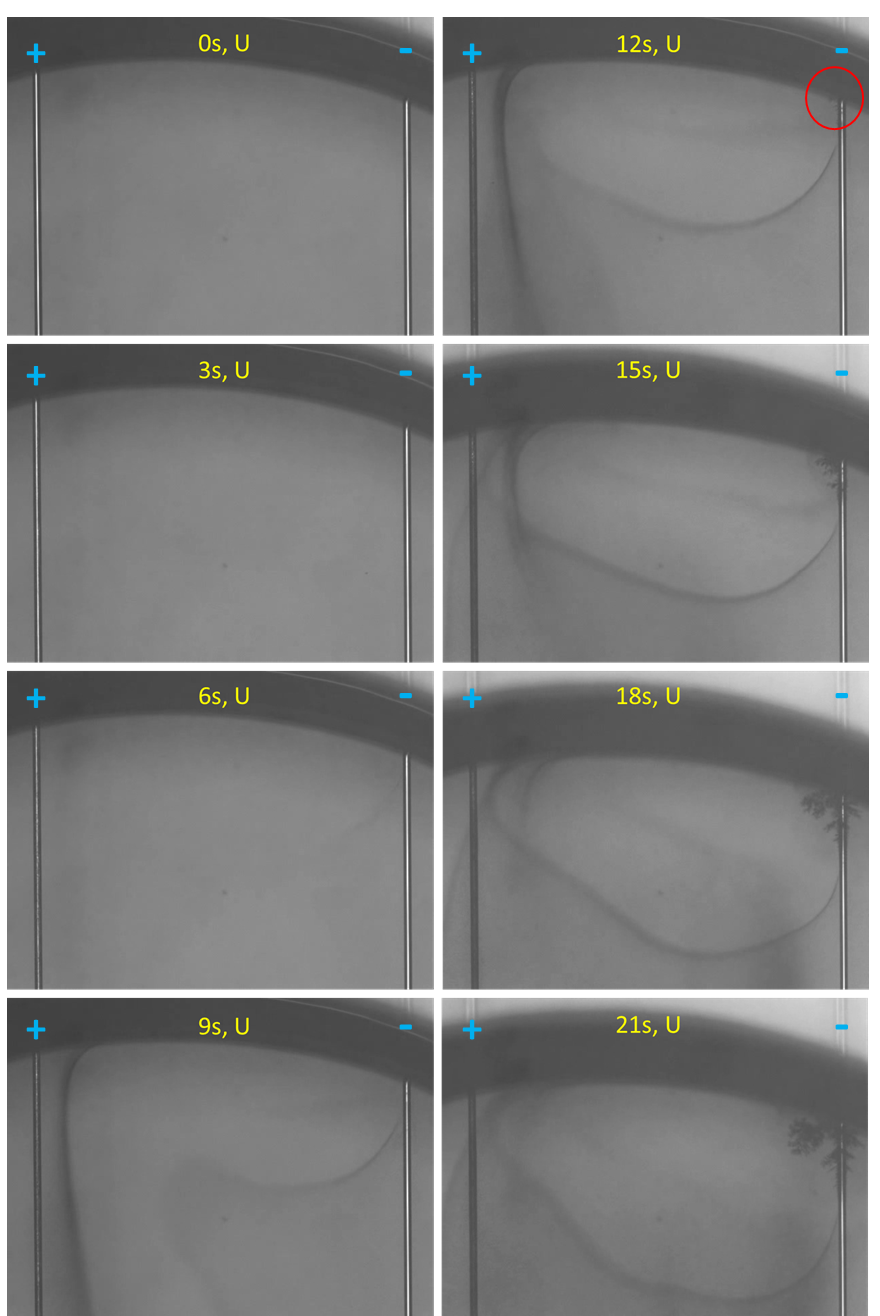


Figure S2 A series of recording frames showing the appearance of a circulation stream after the application of electric potential. The two silver wire electrodes, the anode to the right and the cathode to the left, are shiny at time 0 sec. The anode wire became dull due to oxidation, soon after electric potential was applied, as shown in the 6 sec frame. The upper right region near the cathode wire began showing a particle stream. The stream extended over time, as seen at the frame at 9 sec. The area below the stream curve became darker. The stream grew to a circle after 15 sec., and the upper end of the cathode wire shows attachment of silver dendrite at 12 sec. The dendrite continued to grow during the electrolytic process. The anode wire appeared to be completely dark at 12 sec, while the cathode wire remained shiny throughout the experiment.
